# Depression Outcomes in Adults Attending Family Practice Were Not Improved by Screening, Stepped-Care, or Online CBT during a 12-Week Study when Compared to Controls in a Randomized Trial

**DOI:** 10.3389/fpsyt.2017.00032

**Published:** 2017-03-20

**Authors:** Peter H. Silverstone, Katherine Rittenbach, Victoria Y. M. Suen, Andreia Moretzsohn, Ivor Cribben, Marni Bercov, Andrea Allen, Catherine Pryce, Deena M. Hamza, Michael Trew

**Affiliations:** ^1^Department of Psychiatry, University of Alberta, Edmonton, AB, Canada; ^2^Strategic Clinical Network for Addiction and Mental Health, Alberta Health Services, Edmonton, AB, Canada; ^3^Department of Finance and Statistical Analysis, University of Alberta, Edmonton, AB, Canada

**Keywords:** depression, cognitive behavioral therapy, pathway, suicide, adult, mental illness, family practice, primary care

## Abstract

There is uncertainty regarding possible benefits of screening for depression in family practice, as well as the most effective treatment approach when depression is identified. Here, we examined whether screening patients for depression in primary care, and then treating them with different modalities, was better than treatment-as-usual (TAU) alone. Screening was carried out for depression using the 9-item Patient Health Questionnaire (PHQ-9), with a score of ≥10 indicating significant depressive symptoms. PHQ-9 scores were given to family physicians prior to patients being seen (except for the Control group). Patients (*n* = 1,489) were randomized to one of four groups. Group #1 were controls (*n* = 432) in which PHQ-9 was administered, but results were not shared. Group #2 was screening followed by TAU (*n* = 426). Group #3 was screening followed by both TAU and the opportunity to use an online cognitive behavioral therapy (CBT) treatment program (*n* = 440). Group #4 utilized an evidence-based Stepped-care pathway for depression (*n* = 191, note that this was not available at all clinics). Of the study sample 889 (60%) completed a second PHQ-9 rating at 12 weeks. There were no statistically significant differences in baseline PHQ-9 scores between these groups. Compared to baseline, mean PHQ-9 scores decreased significantly in the depressed patients over 12 weeks, but there were no statistically significant differences between any groups at 12 weeks. Thus, for those who were depressed at baseline Control group (Group #1) scores decreased from 15.3 ± 4.2 to 4.0 ± 2.6 (*p* < 0.001), Screening group (Group #2) scores decreased from 15.5 ± 3.9 to 4.6 ± 3.0 (*p* < 0.001), Online CBT group (Group #3) scores decreased from 15.4 ± 3.8 to 3.4 ± 2.7 (*p* < 0.01), and the Stepped-care pathway group (Group #4) scores decreased from 15.3 ± 3.6 to 5.4 ± 2.8 (*p* < 0.05). In conclusion, these findings from this controlled randomized study do not suggest that using depression screening tools in family practice improves outcomes. They also suggest that much of the depression seen in primary care spontaneously resolves and do not support suggestions that more complex treatment programs or pathways improve depression outcomes in primary care. Replication studies are required due to study limitations.

## Introduction

Depression is recognized as one of the most prevalent and costly conditions in society, occurring in approximately 10–20% of patients attending their family care physicians (1). However, it has been estimated that less than half are adequately recognized and treated (2). Depressed patients have higher rates of morbidity and mortality for a given level of medical illness (3–7). More specifically, depression occurs commonly in patients presenting to primary care physicians (8) and is frequently not diagnosed (9–11). This is important since primary care patients with depression have higher levels of morbidity and mortality, as well as greater health care costs, than similar patients without depression (12, 13). Thus, it is important to identify depression occurring in primary care patients, as well as treat it more effectively.

One suggested method to increase depression recognition is to screen for this in all adults attending primary care (14, 15) and/or looking more intently for the presence of depression in those with specific medical conditions (16, 17). Indeed, a recent US Preventive Services Task Force Recommendation was that screening for depression should occur in primary care for all adults who have not been screened previously (18). To assist this, it has been proposed that standardized depression screening tools be used for this in primary care (19). There are tools designed to help with patients screening, including patient centered-ones (20), and to also assist physicians determine appropriate antidepressants to use (21). One of the most widely used screening tools is the 9-item Patient Health Questionnaire (PHQ-9), a depression screening measure specifically developed for use in primary care (22), and widely validated in primary care (23–25).

However, it should be noted that, in contrast to the US Preventive Services Task Force Recommendation, the Canadian Task Force on Preventive Health Care specifically recommended that screening for depression should not occur in primary care (19, 26). One of the reasons for such conflicting advice may be the relative lack of randomized controlled studies that can help clarify the potential benefits (or otherwise) of screening for depression in primary care. Another possible reason for these opposing recommendations may be the relative lack of access to treatment options for depressed patients when they are identified by primary care physicians.

Additionally, and the major focus of the present study was to try and help clarify the most appropriate treatment option when depression is identified. This includes previous development of a variety of Stepped-care pathways, which have been developed for use in primary care. Stepped-care treatment models usually include both medication and psychosocial interventions and may improve outcomes for depression in primary care (27–33). However, such programs may be difficult to implement, can be resource and staff intensive, and the specific components of individual programs can vary widely (34, 35). Furthermore, not all studies have found statistically significant benefits from Stepped-care pathways (36, 37), and it remains somewhat uncertain which components are most effective. Several primary care practice characteristics can also significantly influence the quality of care provided to patients with depression and comorbid chronic conditions (38). Thus, while it is generally believed that effective treatment of depression decreases subsequent primary care visits (39), the success of any programs may be dependent upon perceptions of the best methods to achieve successful outcomes, and these in turn may differ between primary care physicians and their patients (40).

Nonetheless, despite uncertainty regarding the most efficacious components of a Stepped-care pathway, it is generally accepted that cognitive behavioral therapy (CBT) is an effective component of treatment of depression in primary care (41). One issue with the delivery of CBT has been access to appropriately trained therapists, and the availability of online versions has helped ameliorate this issue. A widely used free online CBT program is MoodGym, and its clinical benefits have been reported in primary care (42, 43), where it has been found to be as acceptable for most patients as face-to-face therapy (44). Such internet-based treatments are scalable and cost-effective (45), potentially making them widely available in primary care (46). Additionally, while it appears that online CBT is effective (47), other therapies may also be effectively delivered online (48). It should be noted, however, that while online psychotherapy programs are useful, they seem to be most effective when combined with face-to-face guided support (49). Indeed, a consistent finding from online studies is that such a “guided” approach usually has much higher retention rates and better outcomes (50–52). Nonetheless, the practicality of a “guided” approach in family practice may be limited because of the resources required.

In summary, current guidelines vary in whether they recommend screening for all adult patients attending a primary care center. Second, there are differences in treatment approaches recommended when primary care patients are identified as being depressed. To enhance understanding in this area, we carried out a double-blind randomized study in which consecutive attendees at family practice sites were offered the option of completing a PHQ-9 rating scale on an electronic tablet while they were waiting for their physician, with follow-up ratings at 6 and 12 weeks for those who were depressed, and 12 weeks only for those who were not depressed at baseline. After informed consent, patients were randomized to one of four groups: (1) control; (2) treatment-as-usual (TAU); (3) online CBT program (MoodGym) (43, 44); and (4) a detailed Stepped-care pathway.

Based on the existing literature, it still remains uncertain whether or not interventions, particularly CBT or a Stepped-care pathway, improved depression outcomes compared to TAU. We are not aware of a previous similar randomized controlled study in primary care. Secondary hypotheses are related to the impact of screening itself and the frequency of onset of new cases of depression during the study period.

## Materials and Methods

### Ethical Consideration

This study was approved by written consent from the Health Research Ethics Review Board at the University of Alberta (Pro00038495) for adults aged 18 who were cognitively capable of giving informed consent. Approval was first given on 30th July 2013 and then included some small changes. The protocol presented in the present paper includes all approved changes.

Note that if any subject expressed suicidal thoughts or feelings at any time, in either written form or verbally, the patient’s physician was immediately notified. This study was conducted according to International standards of Good Clinical Practice (International Conference on Harmonization guidelines), the Declaration of Helsinki (2008 amendment, Seoul, Korea), applicable government regulations and Institutional research policies and procedures. It was registered with Clinical Trials database, http://ClinicalTrials.gov Identifier: NCT01975207.

### Study Flow

**Figure 1:**
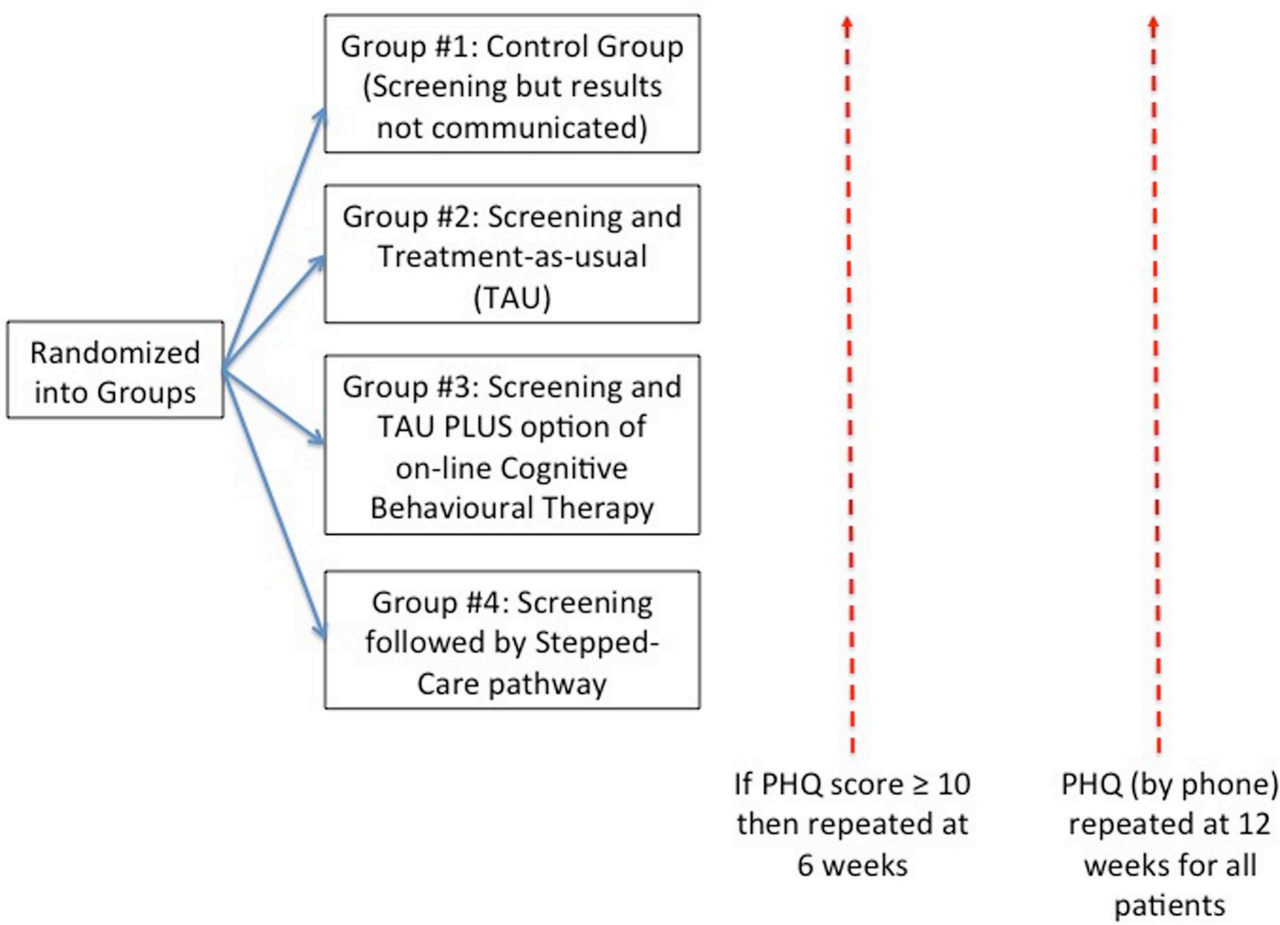
**Patient flow during study**. This shows the flow of patients who were randomized to each of four groups. All patients were followed up by telephone at 12 weeks. For those patients whose score was 10 or more on the 9-item Patient Health Questionnaire (PHQ-9), there was an additional telephone follow-up at 6 weeks.

After a patient had registered for their appointment with their primary care physician, they were informed there was a study taking place and were given information about this. If they expressed interest, they were given information, and if they wanted to proceed they then signed an informed consent form, and also had the opportunity to discuss the study with a member of the study team. Only at this point, they were included in the study, given a unique study number, and were able to complete the PHQ-9 on an electronic tablet (Figure 1).

### Primary Study Hypotheses

The primary study hypothesis was that active treatments for depressed primary care patients (defined as those patients who scored ≥10 on PHQ-9 score following screening) would have lower scores for depression at 12 weeks compared to both controls and those who only received TAU. Thus, it was hypothesized that PHQ-9 scores would decrease to a statistically significant greater degree at 12 weeks in Group #3 (Online CBT Treatment + TAU) and Group #4 (Stepped-care pathway) compared to Group #1 (Screening Control) and Group #2 (TAU). The detailed descriptions of each of these Groups are as follows:

• *Group #1, Screening Control group*: scores were not communicated to the patient or their physician, unless they indicated a suicide risk (utilizing predetermined criteria).
• *Group #2, TAU group*: those who scored ≥10 on the PHQ-9 were informed of this, and the score was documented, and both the patient and their physician were notified of their score. In Group #2 treatment was up to the physician and was TAU.
• *Group #3, Online CBT Treatment + TAU group*: those who scored ≥10 on the PHQ-9 were informed of this, and the score was documented, and both the patient and their physician were notified of their score. In addition to TAU, patients were also informed about a free online CBT program, MoodGym (43, 44). They were encouraged to use it, given a unique log-in number, and were given some information about previous publications suggesting clinical efficacy of this program.
• *Group #4, Stepped-care pathway*: those who scored ≥10 on the PHQ-9 were informed of this, and the score was documented, and both the patient and their physician were notified of their score. In Group #4, treatment followed a specific Stepped-care pathway, in which all clinic physicians and therapists were trained. This was based on current research literature and had been used previously (53, 54). More details of the Stepped-care pathway are given below.

### Secondary Hypotheses

There were additional secondary study hypotheses.

• That screening for depression would lead to lower scores in all groups when the presence of significant depression scores was supplied to physicians (Groups #2, #3, and #4) compared to the controls (Group #1), where this information was not supplied to either the patient or physician.
• That the spontaneous rate of new depression over the 12-week period would be consistent with the existing literature (55).

### PHQ-9 Item

To measure depression, we used the PHQ-9, a specific depression screening measure developed for use in primary care, which has since been widely validated (22–25). A score of ≥10 on the PHQ-9 indicates the presence of clinically significant depressive symptoms. In the present study, those who scored ≥10 on the PHQ-9 were considered depressed. Previously, research has suggested that scores of 10–14 are consistent with mild depression, scores of 14–20 are consistent with moderate depression, and scores of >20 are consistent with severe depression (22–25).

A recent study carried out a meta-analysis, meta-regression, moderator, and sensitivity analysis of screening clinical utility of the PHQ-9 in primary care from over 40 studies involving nearly 30,000 people (56). The authors reported that the sensitivity for the PHQ-9 using a cut-off point of 10 was 81% (95% CI 72–89), and the specificity was 85% (95% CI 81–89). The authors suggested that the PHQ-9 is appropriate for screening but should not be used to confirm a clinical diagnosis.

In the present study, patients completed PHQ-9 questionnaire while waiting to see their physician. All patients were informed they would be followed up by telephone. In the follow-up telephone calls, the PHQ was read to the patient during this phone call and scored according to their answers. A similar approach has been used in previous research (57).

### Stepped-Care Pathway

The Stepped-care pathway used in the present study was previously developed in Calgary, AB, Canada, utilizing updated research evidence and had been successfully utilized in 158 patients in an open-label study in 5 primary care locations during the period 2010–2011 (53, 54). In this previous open-label study, patients who scored 10–14 on the PHQ-9 (*n* = 61) were assigned to the watchful waiting level of intervention, patients who scored 15–19 (*n* = 54) were assigned to the moderate intervention level, and patients who scored 20 or higher (*n* = 43) were assigned to the high intervention level. Successful completion of the pathway was defined as scoring in the non-clinical range (<10) on the PHQ-9. The overall 6-month successful completion rate was 56%, and the mean reduction in PHQ-9 scores was −8.29 (SD = 6.03). At a 3-month follow-up after successful completion of the pathway, 80% of the patients assessed continued to score in the non-clinical range on the PHQ-9 (53, 54). The same methodology was utilized in the current study in Group #4, and training was given to all involved with providing this Stepped-care pathway.

In the present study, for those patients whose PHQ-9 scores were in the range 10–14 there was an initial period of “watchful waiting,” with a set follow-up appointment in 4 weeks and targeted self-management information. There were also specific clinical interventions for those whose scores were 15 or more. This intervention included additional visits, self-management information, medications prescribed according to guidelines, outside referral options, including a psychiatry consultation if they have non-response to medication within 6 weeks. It is important to note that additional resources were provided to those patients who were depressed and were randomized to Group #4, and this included increased availability of cognitive behavioral therapists.

### Data Security and Data Collection

Collection of all data was on dedicated electronic tablets and was compliant with the local and international requirements for data collection. No personal information was collected (including age or gender), only the patient study number and Alberta Health Care Number. The Alberta Health Care Number cannot be linked to an individual, as this information is stored in a separate database to which the investigators had no access. It was collected to allow potential future anonymous analysis of health care utilization. Electronic data were transmitted in an encrypted manner over the internet. As soon as patient data were transmitted, neither the participant nor the study staff had any further access to that (or any other) information. It was not possible for any patient or study personnel to view their information, or anyone else’s until the study was complete. All data were stored in an encrypted manner by an independent organization that was authorized to maintain such information. At the end of the study, anonymized data were available to the research staff to analyze.

### Randomization and Statistical Analysis Plan

Randomization was carried out at both a clinic and day level. There were two clinics involved, and one of these was able to enter patients into Group #4 (Stepped-care pathway). Therefore, in one clinic, patients were randomized to one of four groups (Groups #1–4) whereas in the second clinic, they were only randomized to one of three groups (Groups #1–3). This is the reason that the number of patients entered into Group #4 was lower than in the other three groups. Additionally, the randomization process was not done at an individual level but for an entire day at a clinic. This is because all communication was carried out in the waiting room and we did not want patients to hear about something being offered to one patient, when they would be offered something different. Therefore, the numbers in each group could not be perfectly matched since they depended upon the number of patients who came to a clinic on the days when that particular group was being offered.

For statistical analysis, the change in PHQ-9 was the primary outcome variable. Statistical analysis was carried out in R, version 3.1.0. In a previous study, there was a decrease of only 2 points in the PHQ-9 at 6 weeks and 6 months when no specific treatment was given (58). In comparison, use of the Depression Pathway for treatment in a pilot study led to a mean decrease in PHQ-9 of 10 points (from a mean baseline of 16) in those who completed the Treatment Pathway (53, 54). From pilot data carried out with the Stepped-care pathway, it was anticipated that 70% of patients would take part again at 12 weeks (53, 54). Additionally, based on this pilot research, we estimated that approximately 70% of subjects would have follow-up data.

Therefore, we estimated that in the completer groups, the mean decrease in PHQ-9 scores in the TAU group (Group #2) would be 2 points compared to a mean decrease in PHQ-9 scores in the Treatment Pathway group of 10 points (Group #4). A power analysis suggested that, with a 95% confidence level, the sample size needed was 32 per group. The anticipated number of patients who score at least 10 on the PHQ-9 will therefore need to be 45 in each group, on the assumption that 70% of patients will complete the 12-week assessment period. This would give a completer analysis of 32 patients per group. If 10% of patients were depressed, this would require a total recruitment of 450 in each of the four groups, for a total recruitment of 1,800 during the study period, which therefore was the number of patients we targeted for recruitment.

Analysis was carried out utilizing Wilcoxon rank paired tests comparing median scores. The Wilcoxon signed-rank test was used to test within group comparisons while the Wilcoxon rank-sum test was used to test between group medians. All results compared baseline scores to the scores at 12 weeks.

## Results

**Figure 2:**
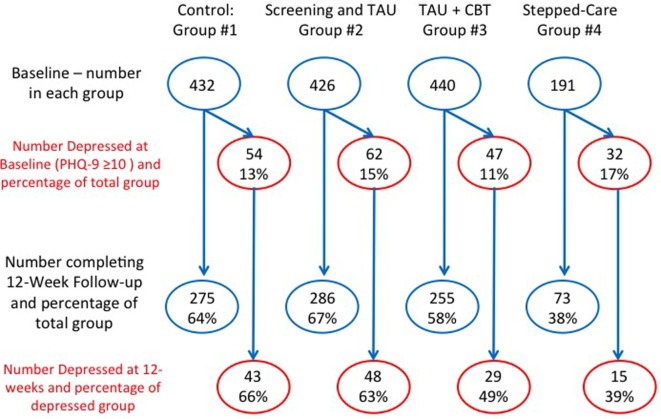
**Patient numbers in each group during study**. This shows the number of patients randomized to each of four groups, and the number who were followed up at 12 weeks (and the percentage) for both the total group and those who were depressed. Group #1 was the Control group; Group #2 was screening followed by treatment-as-usual (TAU); Group #3 was screening followed by TAU and online cognitive behavioral therapy (CBT); and Group #4 was the screening followed by Stepped-care. Note that not all clinics offered the Stepped-care option that is why fewer patients were randomized to this group.

This study was carried out at two different clinics containing a total of 18 primary care physicians. During the study recruitment period from November 2013 to December 2014, a total of 1,489 patients were recruited into the study. Because patients would frequently attend several times at the same clinic during the 6-week periods we spent at each clinic, we were not able to measure the actual number of patients who were potentially eligible for the study. As noted previously, as only one of the clinics agreed to take part in the Stepped-care pathway, the numbers of patients randomized to the Stepped-care pathway (Group #4) were lower than the other groups. A total of 432 patients were randomized to the Control group (Group #1), 426 patients were randomized to screening followed by TAU (Group #2), 440 patients were randomized to TAU plus online CBT (Group #3), and 191 were randomized to the Stepped-care pathway (Group #4). Of these patients, approximately 15% of each group were depressed at baseline (PHQ-9 score ≥10) (Figure 2). A total of 889 patients (60%) of patients had a follow-up at 12 weeks, although this percentage varied between groups (Figure 2). Although not reported separately, the results did not change when controlling for site.

In terms of depression outcomes, at baseline there were 54 (10.0%) depressed patients in the Control group, 48 (11.3%) depressed patients in the Screening group, 29 (6.6%) depressed patients in the Online CBT group, and 15 (7.9%) depressed patients in the Stepped-care pathway. There were no statistically significant differences in baseline PHQ-9 scores between these groups of depressed patients.

**Figure 3:**
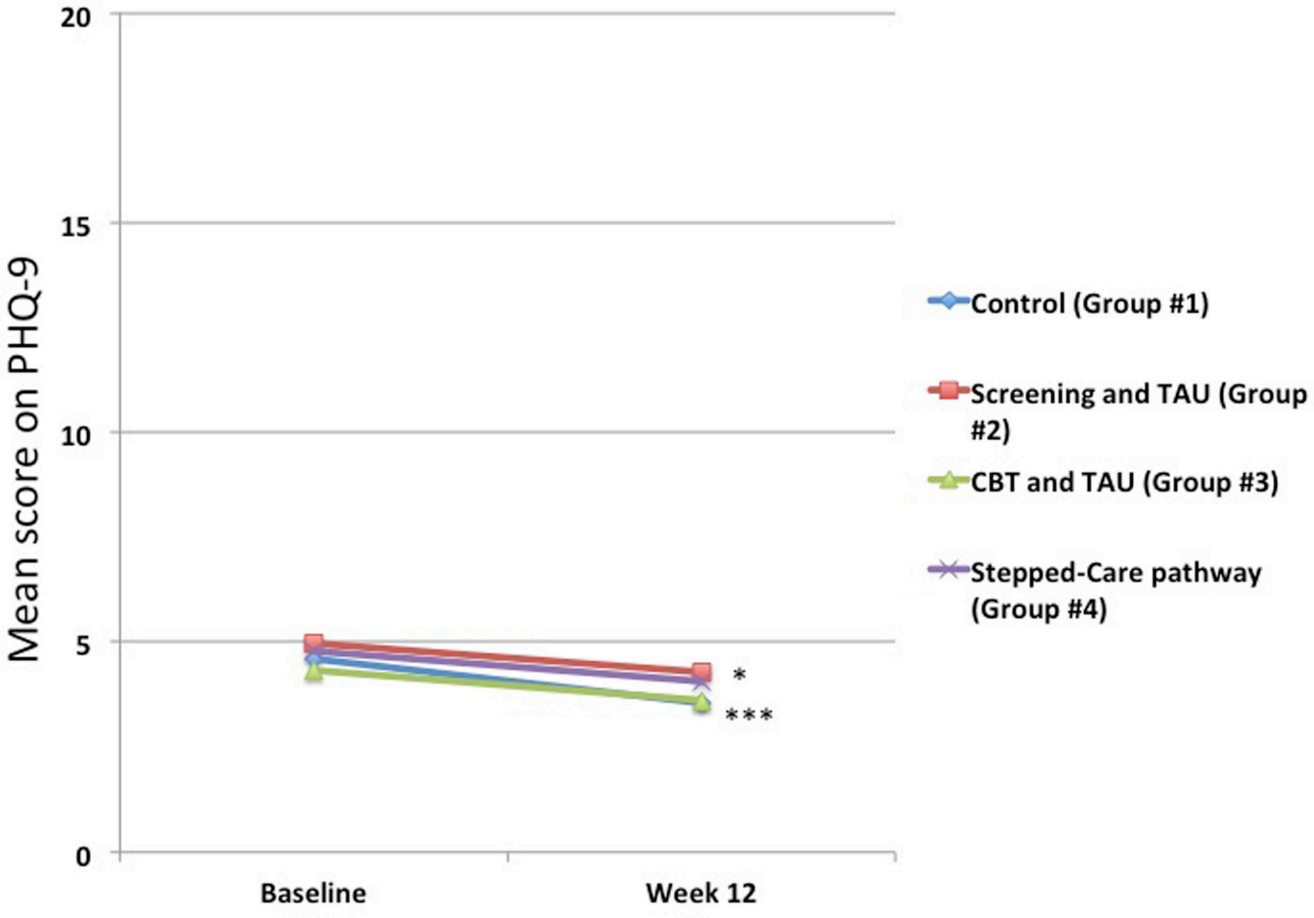
**Changes in mean 9-item Patient Health Questionnaire (PHQ-9) scores for total group**. The mean scores from baseline to 12 weeks decreased across the entire group, but these were only statistically significant for the Control Group #1 (***p < 0.001) and the Screening and Treatment-as-usual Group #2 (*p < 0.05). There were no statistically significant changes for either Group #3 or Group #4. The number of patients in each group at both baseline and 12 weeks is shown in Figure 1.

For all of the groups, there were statistically significant changes in PHQ-9 scores from baseline to 12 weeks (Figure 3) for Groups #1 and #2, but not for the treatment interventions, Groups #3 and #4. Thus, in the Control group (Group #1), this changed from a mean score of 4.6 ± 5.0 to 3.6 ± 4.3 (*p* < 0.001), and in the Screening group (Group #2), this changed from a mean score of 4.8 ± 4.9 to 4.3 ± 4.7 (*p* < 0.05). In the online CBT and treatment-as usual group (Group #3), this changed from a mean score of 4.1 ± 4.4 to 3.6 ± 4.4 (*p* = 0.06), and in the smaller Stepped-care group (Group #4), this changed from a mean score of 4.8 ± 5.5 to 4.1 ± 4.9 (*p* = 0.27).

**Figure 4:**
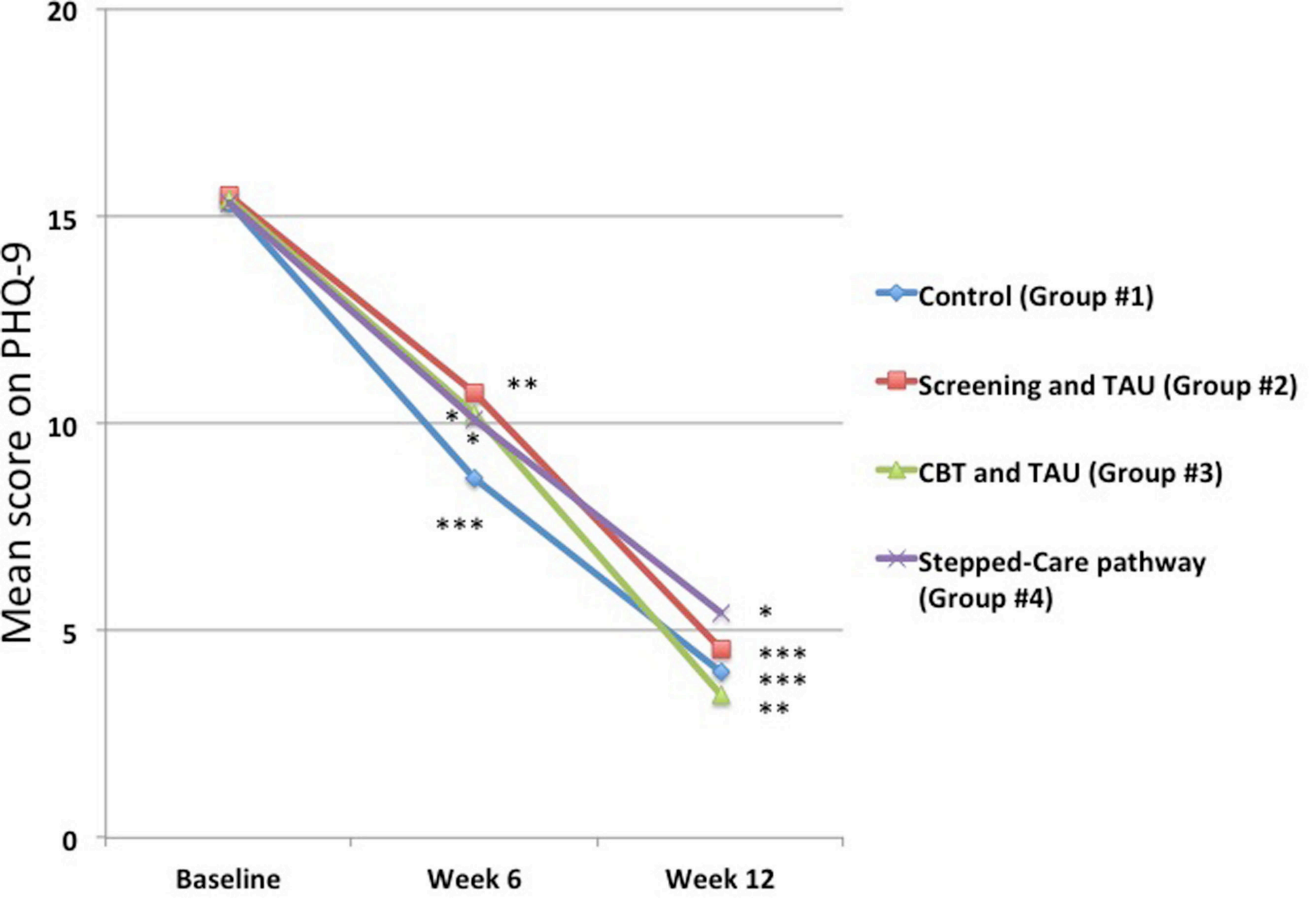
**Changes in mean 9-item Patient Health Questionnaire-9 (PHQ-9) scores for those patients who were depressed at baseline**. It can be seen that the mean PHQ-9 scores decreased significantly at both 6 and 12 weeks in all groups. However, there were no statistically significant differences between any of the groups at either time point. *p < 0.05 compared to baseline, **p < 0.01 compared to baseline, ***p < 0.001 compared to baseline.

In contrast, there were marked changes in mean PHQ-9 scores for those who were depressed at baseline. While the results showed that there were no statistically significantly differences at baseline between the four groups in terms of the means scores for those who had a PHQ-9 score ≥10 (Figure 4), in all groups there was a very marked drop in mean PHQ-9 scores at both 6 and 12 weeks (Figure 4). Control group (Group #1) scores decreased from 15.3 ± 4.2 to 4.0 ± 2.6 (*p* < 0.001), Screening group (Group #2) scores decreased from 15.5 ± 3.9 to 4.6 ± 3.0 (*p* < 0.001), Online CBT group (Group #3) scores decreased from 15.4 ± 3.8 to 3.4 ± 2.7 (*p* < 0.01), and the Stepped-care pathway group (Group #4) scores decreased from 15.3 ± 3.6 to 5.4 ± 2.8 (*p* < 0.05). However, there were no statistically significant differences between any of the groups in the amount of change in PHQ-9 scores at either 6 or 12 weeks (Figure 4).

It should also be noted that among the 889 patients who completed both baseline ratings and again at 12 weeks, a total of 21 individuals had PHQ-9 scores ≥10, but whose scores were less than 10 at baseline. This would suggest that there was an incidence rate of depression of 2.4% over 12 weeks (an approximately annual incidence rate of 10%) in this sample.

## Discussion

We are not aware of another randomized study in primary care that has controlled for screening in the same manner as we have done. This is important to note, as the findings from our study do not support suggestions that screening for depression in primary care improves outcomes, at least as measured by PHQ-9 depression scores. While this was not the goal of the study, or part of our hypotheses, it is important to note as it has significant implications. Additionally, the present findings show that in the vast majority of cases of depression in primary care resolution occurs over a 12-week period, even in the absence of screening information passed onto the patient of physician to identify the presence of depression. Our other finding was that the type of intervention also appeared to make little difference and does not support the use of complex Stepped-care pathways compared to usual care. However, the clinics that took part were self-selected, and it is conceivable that these primary care practices practice an evidence-based approach and have a high degree of awareness for the risk of depression occurring in their patients, and thus screening would be unlikely to identify additional patients and many of the physicians may already be carrying out best practices. Additionally, as we did not have access to the clinical charts, we could not determine whether or not patients who were depressed in the Control group were in fact already detected by physicians.

One other point to note was that in reality there was very little difference between Group #2 (screening followed by TAU) and Group #3 (screening followed by TAU but with the addition of online CBT). This is because, despite our best efforts, uptake of the online CBT was very low. While a total of 25 of the potential 29 depressed patients in this group logged on, less than 5 completed more than 1 CBT session, and none of the patients completed the entire program. Thus, the possible potential impact of the CBT approach was limited. These rather disappointing results are similar to others, which have found that unless there is “guided” CBT (i.e., a person encouraging an individual on a repeated basis) very few individuals complete online CBT programs for depression (43, 49–52).

In terms of improvements, it is well recognized that many patients who have depression in primary care improve spontaneously, with one review estimating that 23% of untreated depression patients in primary care will remit within 12 weeks, with higher remission rates occurring in those who have milder depressive illness (59). The fact that the mean PHQ-9 scores in all the groups were quite low at baseline (13.7–14.5) would be consistent with this.

### Other Methodological Issues and Study Limitations

Despite the fact that this is a randomized controlled study, there were significant limitations that require further research before any definitive recommendations can be made. The first issue is that this study took place in only two clinics, and not the five we had intended. A second concern is that, particularly in the clinic who had training on the Stepped-care pathway, they could have integrated many of those approaches to their practice as part of their TAU, thus decreasing the apparent impact of the Stepped-care approach. Another issue with the data is that not only was the Stepped-care group much smaller than the others but it also had by far the lowest retention rates for the study. We are uncertain why this is the case, but it is possible that those who did not have a 12-week follow-up were doing better than those who were followed up. It is also important to note that our goal was to recruit patients from five separate clinics. However, despite approaching multiple clinics within the region we were able to only involve two clinics, only one of which was willing to take part in the Stepped-care pathway. This is why there were fewer patients randomized to this group (Group #4). Reasons from potential clinics as to why they were unwilling to take part in the research study included the following:

• Concerns about clinic staff resources being required was a major worry for many clinics. Many clinicians were also concerned that taking part in the study would interfere with the flow of patients.
• Clinics felt that their mental health teams were already too busy and were concerned the study would identify more patients needing treatment. They felt they could not manage more referrals, even if it was seen as a chance to prevent depression from worsening.
• Clinics were burdened with other priorities including other multiple quality improvement initiatives that were prioritized by clinic staff.
• Concerns around managing information on an ongoing basis was a concern, particularly as larger clinics have several physicians who may interact with the patient and can be hard to standardize the care approach

There were also specific concerns expressed about the Stepped-care pathway:

• The Stepped-care pathway requires initial and ongoing training—seen as time intensive for staff that are already busy with many priorities
• Stepped-care pathway requires repeated use of standardized tools and approaches, including specific advice regarding which medications would be used for specific patients, thus decreasing physician independence.
• Clinics felt that new evidence was always coming out, making it difficult to keep a detailed Stepped-care pathway up-to-date.

It should also be noted that these were “convenience” samples, i.e., only patients who came to the family practice were eligible. In terms of the randomization approach, since the waiting areas were open, it was likely that other patients in the waiting room would be aware of the study. For this reason, it was determined that randomization should occur per day, and not per patient. Thus, all patients who attended a specific clinic on an individual day would all be entered into the same group. This would avoid any possible issues where one patient in the waiting room, for example, was offered the opportunity to take part in online CBT while another was not. Another aspect that needs consideration is that after a period of time (usually 4–6 weeks) many of the patients coming to a specific clinic were those who had already come in the previous period, and therefore were not eligible. For this reason, recruitment rates decreased over time at each clinic and to address this recruitment alternated between clinics for a 6-week period (i.e., patients were only recruited at specific clinics for 6-week periods, before returning to the same clinic after a similar time period). Additionally, we did not have the resources to examine the medical charts of the patients who took part in the study. Therefore, we were not able to compare medication use, actual diagnosis, other medical diagnosis, or have access to demographic data. All of these may have helped understand the study population in more detail.

It can also be seen that making patients aware of the study, completing the informed consent process, and then completing the forms, took time. There were therefore many patients who expressed willingness to take part, and who started to complete the PHQ-9, but before completion they were called in for their appointment. In all such cases, the patient stopped entering data immediately and was not included in the study.

In terms of follow-up calls, our protocol only allowed an initial call and a maximum of only two follow-up calls, which all had to be carried out over a few days. It is possible, therefore, that different calling protocol, or use of other communication tools, may have led to higher follow-up rates at 12 weeks.

## Conclusion

Despite the methodological issues, the present study was carried out in a relatively large group of patients who were then followed up for a meaningful period. Despite this, there was no evidence suggesting either that screening enhanced depression outcomes, or that any specific treatment intervention was more effective than TAU. Supporting the relevance of the study, our findings that the annual incidence for depression was approximately 10% are consistent with some other studies (55), although one recent study found an incidence rate of only 5% (60).

In conclusion, the findings from the present randomized controlled study in family practice suggest that most patients who have depressive symptoms have mild depression, much of which will resolve spontaneously. Our findings did not support any additional benefit for screening of patients for depression, or the use of specific treatment approaches when compared to TAU. Nonetheless, given the major burden of depression, its impact upon medical health as well as psychological health, and the key desire to minimize its occurrence, as well as the limitations of the present research, further randomized well controlled research studies in this area are critical.

## Author Contributions

PS, KR, VS, MB, AA, CP, and MT designed and supervised this study. AM and DH were involved in managing and running the study and project management and data collection. IC and VS were involved in data analysis.

## Conflict of Interest Statement

The authors declare that the research was conducted in the absence of any commercial or financial relationships that could be construed as a potential conflict of interest. The reviewer NY and handling editor declared their shared affiliation, and the handling editor states that the process nevertheless met the standards of a fair and objective review.
